# Developing ophthalmology in Cambodia

**DOI:** 10.1038/s41433-023-02846-9

**Published:** 2023-12-22

**Authors:** Priyanka Mandal, Camrun Shah, Sunil Shah

**Affiliations:** 1 Department of Ophthalmology, University Hospitals of Coventry and Warwickshire NHS Trust, Coventry, UK; 2 University of Exeter Medical School, Exeter, UK; 3 College of Health and Life Sciences, Aston University, Birmingham, UK; 4 Midland Eye, Solihull, UK

**Keywords:** Health care, Diseases

## Abstract

Over 180,000 Cambodian people are blind and a further 10,000 suffer avoidable blindness each year. Ninety percent of this blindness is avoidable, 79% is curable and 11% is preventable. Three-quarters of this blindness is due to cataracts and the remainder is due to uncorrected vision, glaucoma, corneal scarring and pterygium. The Khmer Sight Foundation (KSF) is a charity reincarnated by Professor Sunil Shah and Sean Ngu. Its mission is to deliver a sustainable eye care model for the country. KSF takes a three pronged approach to this. It is working to develop sustainable eye-care within the country through building physical infrastructure. The second approach is to impact the current cataract backlog of over 300,000 patients with the aid of international support. Thirdly, KSF is paving the way for the next generation through the development of an optometry education programme and training of Cambodian ophthalmologists. Here we present the workings of KSF, clinical cases we have encountered and elaborate upon the future goals of this charity.

## Abstract (zh)

柬埔寨每年超过18万人失明, 另有1万人患有可避免盲。其中90%的失明是可以避免的, 79%是可以治愈的, 11%是可以预防的。四分之三的失明是由于白内障导致的, 其余是由屈光不正、青光眼、角膜瘢痕和翼状胬肉等疾病造成的。Khmer视觉基金会(KSF)是由Sunil Shah教授和Sean Ngu教授创立的慈善机构。其宗旨是为国家提供可持续的眼部护理模式。KSF对此采取了三管齐下的方法, 通过建设有形基础设施在国内发展可持续的眼部护理。第二种方法是在国际的支持帮助下, 解决目前积压的30多万白内障患者。第三, KSF通过开展验光教育计划和培训柬埔寨眼科医生为下一代的成长铺路。在此, 我们介绍了KSF的运作模式和遇到的临床案例, 并阐述了该慈善机构的未来目标。

## Introduction

It is an honour to deliver this eponymous lecture in honour of Professor Barrie Jones. Professor Jones was originally from New Zealand and became the first Professor of Ophthalmology at Moorfields. He was a distinguished Ophthalmologist with a prestigious legacy in research-based medicine. Professor Jones received many honours during his lifetime of work, including a CBE, and his main interests were around infectious diseases - in particular, trachoma [1]. This current piece is therefore befitting of the humanitarian nature of Professor Jones’ work.

My involvement in the Khmer Sight Foundation (KSF) began when I was introduced to Sean Ngu through my social networks in 2016. At the time, Sean was one of the Secretaries of State for Cambodia and a humanitarian. Sean himself had been a refugee from the Khmer Rouge. He was brought up in Australia and came back to Cambodia as an adult to help rebuild the country.

Sean was well aware of the prevalence of eye disease in Cambodia and the many ways this impacts upon a person’s life. Thus, the KSF was born. Its mission is to deliver a sustainable eye care model for the country.

We discuss how KSF has helped developed eye services in Cambodia, a lower middle-income country where eye health services are unaffordable and inaccessible to those in need.

## The problem

Cambodia is a country with a tragic recent history. During the 1970s, the country faced mass genocide under the rule of the Khmer Rouge. The organisation targeted anyone suspected to be an enemy of the regime which led to the execution or exodus of professionals, intellectuals and the educated [2]. Today, Cambodia has one of the lowest number of ophthalmologists per capita in the world - there are only 23 surgically trained ophthalmologists for this country of over 16 million people. There are no fully trained optometrists and no trained ophthalmic nurses.

Over 180,000 Cambodian people are blind and a further 10,000 suffer avoidable blindness each year. Ninety percent of this blindness is avoidable, 79% is curable and 11% is preventable. The majority of this visual loss is due to cataracts and the remainder is due to uncorrected vision, glaucoma, corneal scarring and pterygium [3].

Almost 20% of the population live below the poverty line with the majority earning less than USD $2 per day [4]. Most of the population live in rural areas with zero or limited access to eye care. Women are more than twice as likely as men to suffer from cataract-related blindness [5] and the known current cataract backlog is over 300,000 - however the majority of patients never see eye care professionals.

Kim Frumar, an Australian Ophthalmologist, had started a Cambodian charity aiming to help solve this problem by undertaking surgical charity missions for one week every year. Unfortunately, Dr Frumar suddenly passed away in 2016 which left this charitable endeavour at a loose end.

## The solution

Sean Ngu and I restarted KSF. KSF’s aim was to enable clinicians to work for a charitable cause where skill sets can be used in an environment free from political interference - a difficult feat considering the countries where eye health inequality is at its worst are often those most likely to have deep-set political biases. Due to Sean’s links with parliament, many political problems have been kept at bay.

Recently, the charity has been adopted by the King of Cambodia as a Royal charity therefore even if the political scene changes, the charity is protected. The Prince and Princess of Cambodia are also on the Board of the charity. It is these and other amazing partnerships that enabled the charity to exist as does is today.

The KSF takes a three-pronged approach to tackle the issue of blindness in Cambodia.

### Developing sustainable eye-care

The charity aims to build local capacity through training local doctors and health workers, building new facilities, and introducing the latest technology and equipment. KSF is helping to create a self-sustaining local centre of excellence for eye care training and service delivery in Cambodia’s capital Phnom Penh. This will enable the creation of a one-stop centre for eye screening and operations. By training nurses, optometrists and ophthalmologists there will be a generation of local specialists ready to take on the challenge of eradicating avoidable blindness.

### Blindness in Cambodia can be impacted very quickly with international support

KSF aims to run ongoing surgical treatment with the help of international doctors to help tackle this backlog of blindness. So far, over 100 UK ophthalmologists and over 250 eyecare professionals have come to Cambodia to work with KSF. Thanks to our social and professional networks, the charity has benefitted from help from international clinics - having fundraised to send patients from Cambodia abroad for treatment where KSF cannot offer the appropriate treatment or support. KSF has also benefited from a huge amount of international support from ophthalmic companies such as Zeiss (Oberkochen, Germany), Rayner (Worthing, West Sussex), Lenstec (Florida, United States), Teleon (Spankeren, Netherlands) and Thea (Clermont-Ferrand, France) in their donations of clinical and surgical equipment. In addition, many of the surgeons have donated unused equipment from their hospitals as well as offering their time on repeated occasions. For example, Florian Kretz, an ophthalmic surgeon from Germany, is one of the doctors who has become very committed to this cause with annual visits (often with an entire clinical team and family) and very generous donations of equipment.

### Paving the way for the next generation

The future depends upon education. There is very little ophthalmology training and no optometry education. As a consequence, there is no ophthalmic nurse training either. KSF are working to create an optometry training program, the first of its kind in Cambodia, as well as increase the number of ophthalmology trainee doctors.

## Screening

**Fig. 1:**
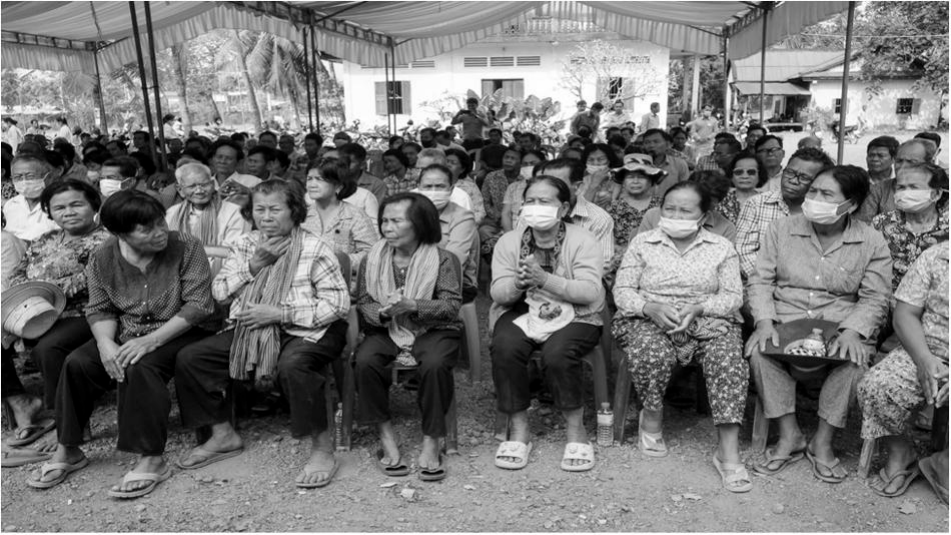
Local screening sessions held in a remote village.

Screening takes place locally in rural villages, where 85% of the population of Cambodia reside. This is a mammoth task in its own right - arranging a team to go out during dry seasons as the roads are impassable during the rainy season. As shown in Fig. 1, these screening sessions signify the calm before the storm; district governors are contacted by Sean from parliament and the local people set it up with appropriate seating for all those that feel they have an eye issue to be seen to. The patients are very friendly and patient no matter how long they sit and wait. They are happy that someone is going to take the time to examine them. Many patients also have non-ophthalmological and complex conditions that are unable to be managed by the team in Phnom Penh.

The screening team consists of local volunteers - students from international schools and one of the Cambodian universities (University of Puthisastra) which encourages their medical, pharmacy and nursing staff to volunteer. Volunteers also act as translators at the interface between patient and clinician, translating from Khmer to English.

Patients have their intraocular pressures taken with an iCare tonometer, their pupils are checked and visual acuity performed. These basic skills are taught to the non-ophthalmic volunteers at screening and patients are then dilated if appropriate for examination, streamlining the use of resources and enabling swift decision making.

If a patient is identified as having a treatable condition or a condition that warrants further review, an electronic patient record (EPR) system entry is created. KSF have instituted a robust system to do this. The charity started working with L V Prasad Eye Hospital in Hyderabad and have a very good EPR to keep track of such high volumes. We instituted this system early so as we expand we can keep track of everybody. This EPR also holds important data for further research.

## The Phnom Penh Centre

Patients arrive on minibuses arranged by KSF. This is often the first time a patient travels outside of their village and bringing them to Phnom Penh, a city of 2 million people, can be a considerable culture shock. Patients are then seen pre-operatively by a member of the local or international team of clinicians. This part of the team is largely comprised of optometrists and junior doctors. Volunteers act as translators. During the counselling process many patients reveal that they feel their eye condition has been given to them from God and some therefore refuse surgery despite screening and travelling a long distance to the clinic.

**Fig. 2:**
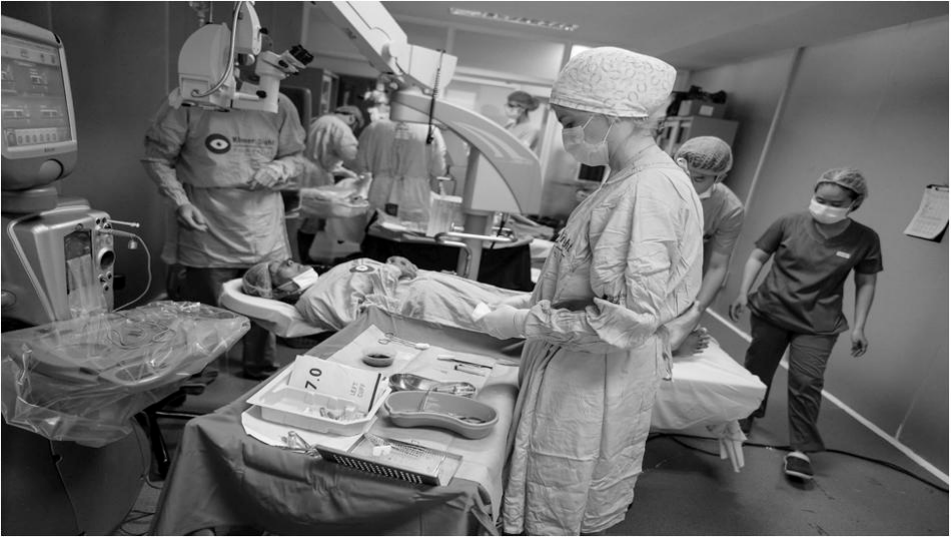
Multiple operating tables running at once.

Anaesthetic blocks are delivered by optometrists trained by KSF clinicians and junior ophthalmology doctors. As shown in Fig. 2, KSF run multiple theatre beds simultaneously. Despite inadequate ventilation (no clean air) compared to the UK, KSF have only had 2 cases of reported endophthalmitis in 26,000 patients. Post surgery antibiotics consist of intracameral cefuroxime or moxifloxacin and perhaps this is a testament to their value.

KSF have high quality equipment such as a Zeiss IOLMaster 700 and old Alcon Infiniti phacoemulsification machines which are serviced every year by a volunteer who works as a phaco technician. The microscopes are also of good quality. The aim is to make the surgeons feel as comfortable as possible as the surgery itself is very challenging.

Patients have their operation and usually stay overnight in the city, arranged by KSF, for a next day post-operative check. They are then taken back to their villages on a minibus and reviewed locally 6 weeks later.

**Fig. 3:**
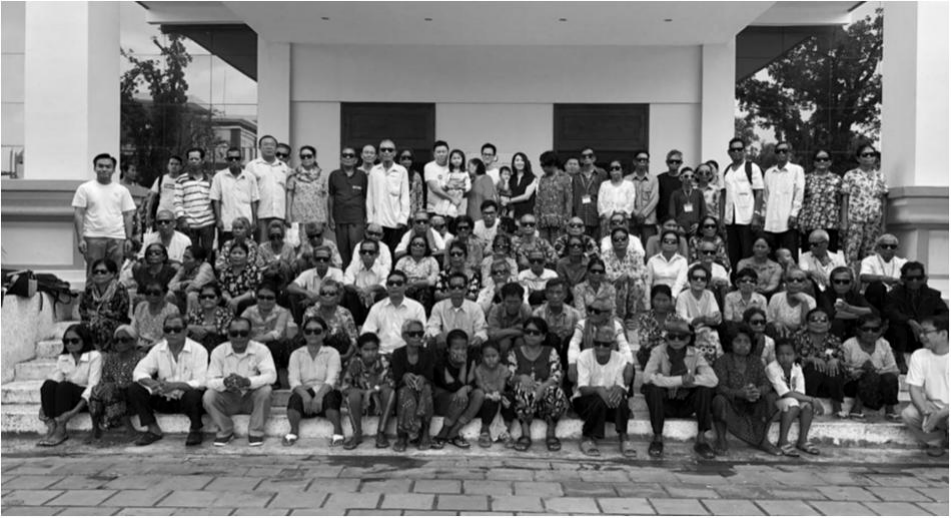
Post-operative patients at the end of the day with their sunglasses.

Juniors, optometrists and friends are all useful as volunteers. Non-medical friends often want to come - they are always able to help even if it is as simple as crowd control. Patient flow and strong organisation is vital as there are so many patients flowing through the process each day. Patients are extremely grateful and love their free post-operative sunglasses as shown in Fig. 3.

**Fig. 4:**
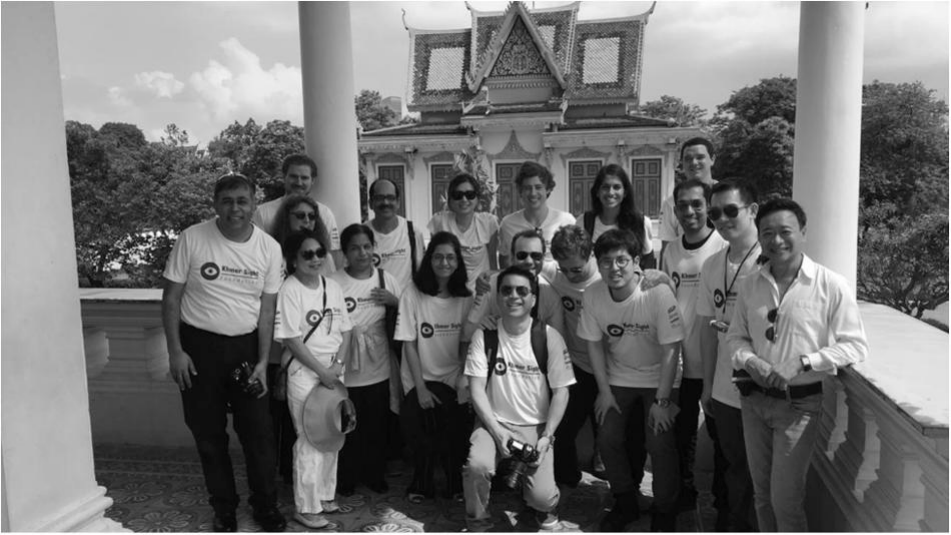
The team on a tour of the palace with Prince Tesso of Cambodia.

Some days can run late depending on the number of cases and severity of disease however we do try and finish early. The volunteers really enjoy the experience. In the evenings the team go out for dinner together and socialise as well as enjoy interesting social downtime such as tours of the Royal Palace with Prince Tesso of Cambodia (Fig. 4).

## Clinical cases

### Orbital and extraocular

**Fig. 5:**
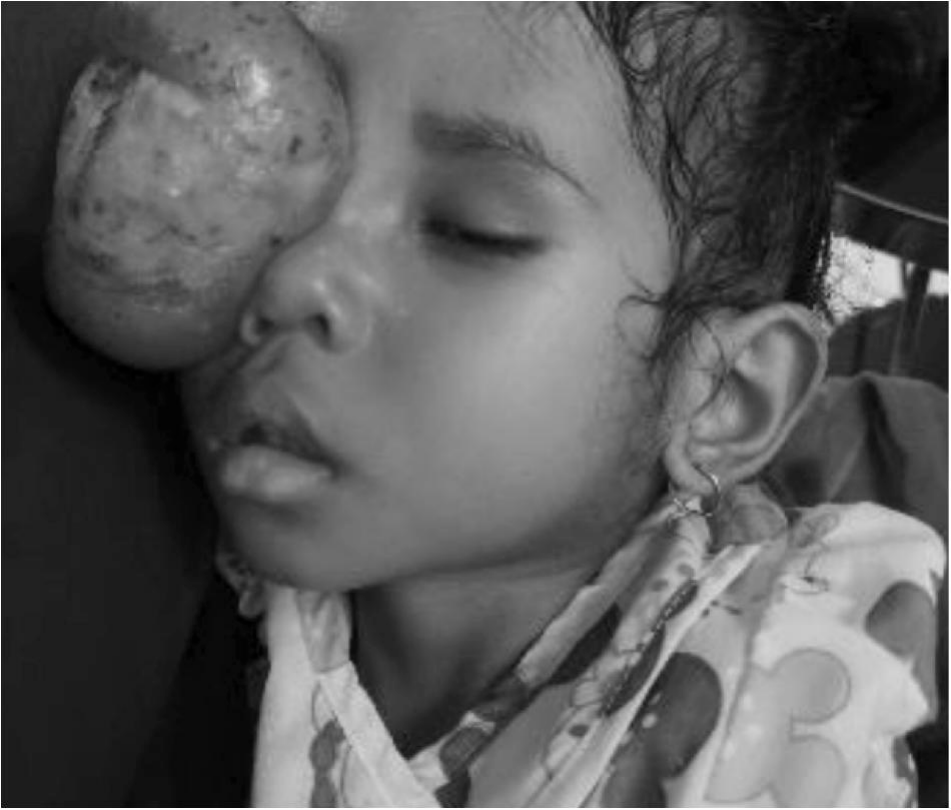
2 year old with an advanced orbital tumour.

The pathology encountered is advanced and therefore not always treatable by KSF. Figure 5 shows a 2 year old with an advanced orbital tumour who was brought to us by her parents as she was unable to access any care. The child died 2 days later. In the UK, losing a patient as an ophthalmologist is rare occurrence in day to day clinical work.

Astoundingly, the parents were so full of gratitude despite being unable to save their daughter. They came the following day and said thank you for caring enough to try and for making her comfortable. Though unable to medically treat this patient, exercising true empathy and care in these cases is invaluable. Clinicians are always prepared to try their best even if the pathology is something that cannot be treated.

**Fig. 6:**
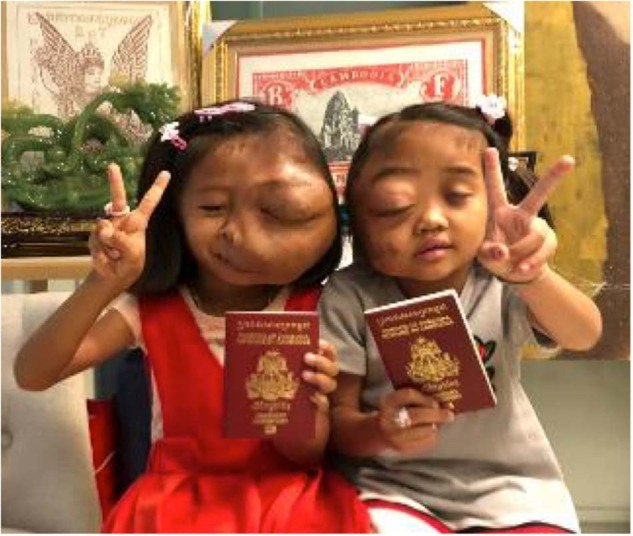
Two girls with severe neurofibromatosis.

Figure 6 shows two girls, not related, with severe neurofibromatosis. These two patients attended the clinic on the same day. The child in red had such severe neurofibromatosis that it had eroded her skull. These little girls were so happy to find someone else like them - they had no friends as they were deemed disfigured by their local communities.

As ophthalmologists, any management of this condition is well beyond our scope. However, KSF arranged a CT scan to be performed locally. As these cases were too risky for Cambodian hospital care, the charity undertook separate fundraising for them and managed to secure passports to fly the patients to Brisbane for treatment.

This is an example of how KSF clinicians have to think laterally and reach out to international colleagues for help. It is also an example of the logistical mapping necessitated; each child was sent with one parent as the other parent had to stay in Cambodia with their other children. They had never left their village before and suddenly they were sent to a completely different country with a different language and culture. The children were in Brisbane for three months. Both of these children have done extremely well.

**Fig. 7:**
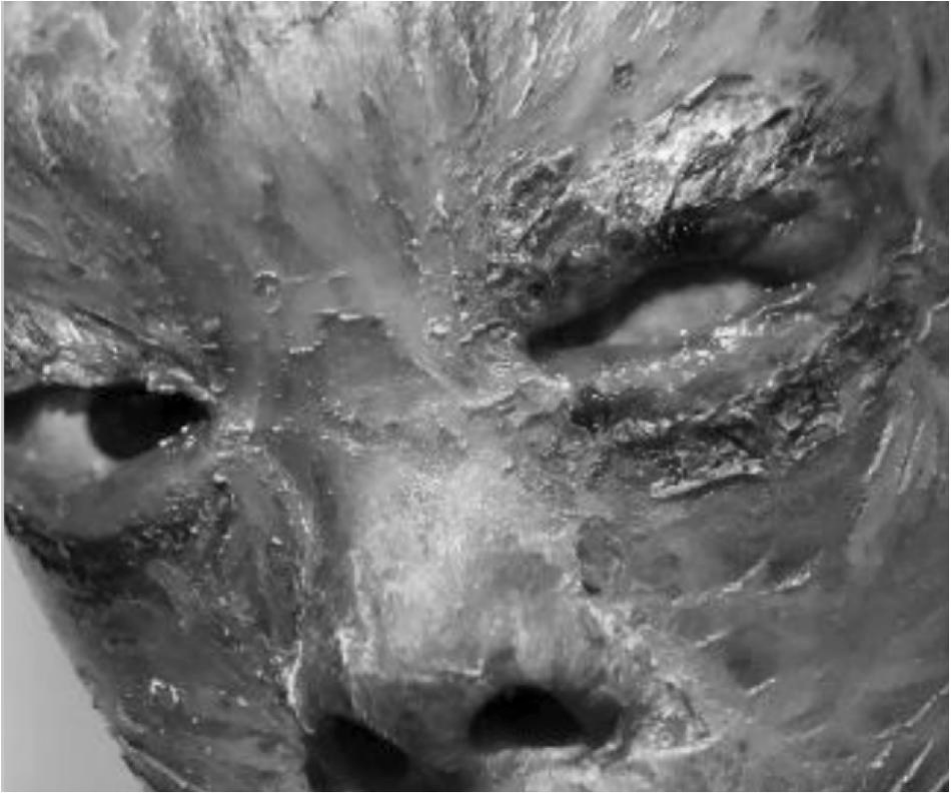
Little boy with congenital ichthyosis.

The little boy shown in Fig. 7 had congenital ichthyosis. His monk had told him that this condition was given from God therefore he should not have any treatment. This is an example of the challenges presented by cultural issues. Here, KSF clinicians worked to protect the other eye.

**Fig. 8:**
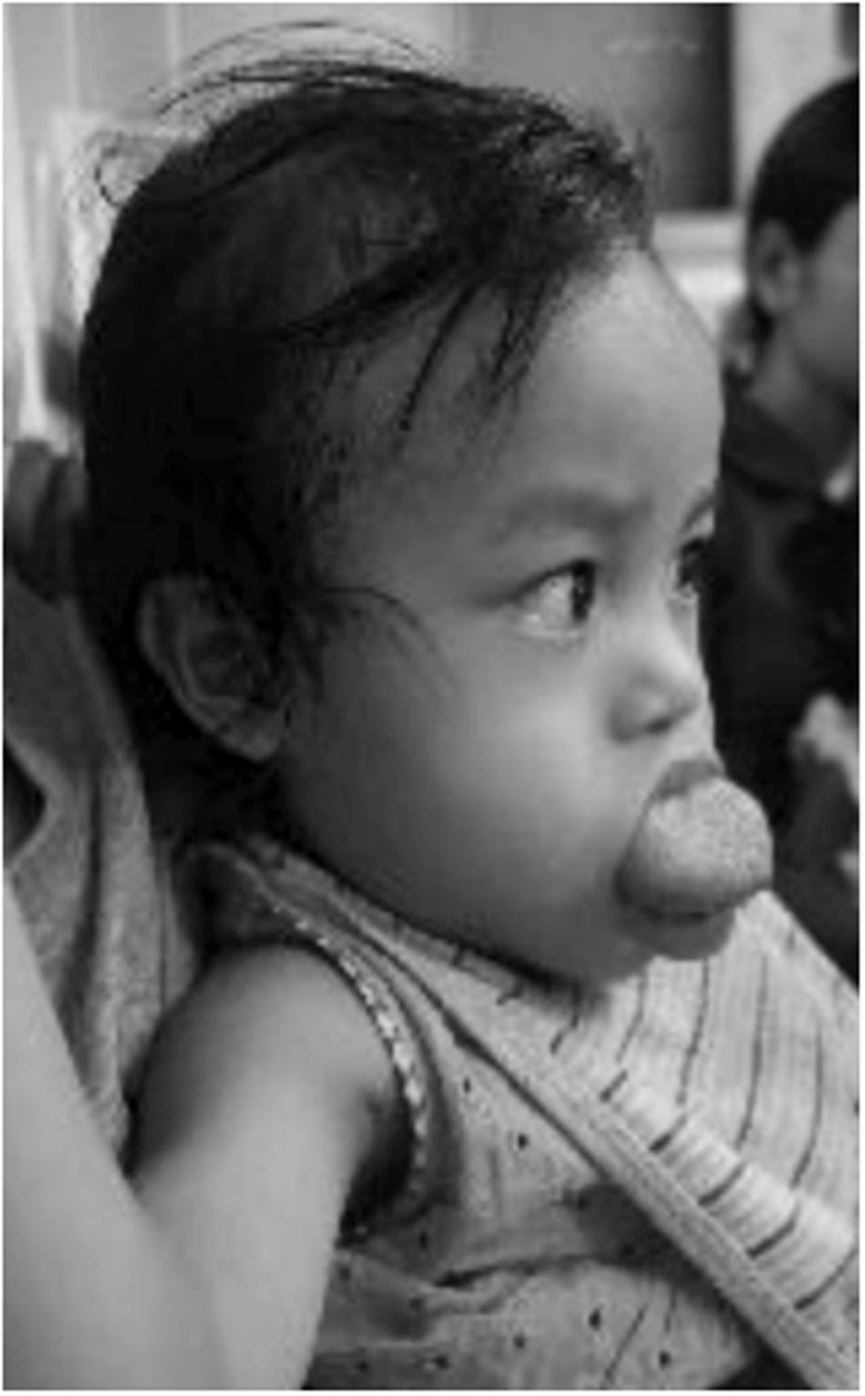
Vascular malformation of the tongue.

The child in Fig. 8 had a vascular malformation of the tongue. She was sent to India for embolisation. Again, an example of having international colleagues and finding people around the world who would be willing to help proved crucial to a successful outcome.

These four patients came to the clinic on the same day - such is the breadth of patients seen by what is called an ‘eye charity’.

### Ophthalmic

**Fig. 9:**
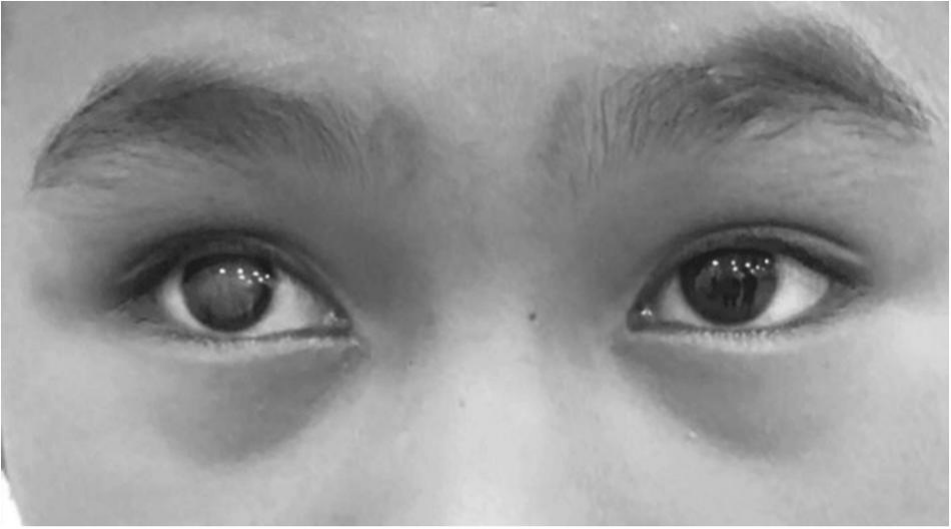
11 year-old girl with a dense white cataract.

We encounter many young patients with cataracts - these may be congenital or secondary to trauma. Figure 9 shows a young 11 year-old girl with a dense white cataract and no fundal view. Upon cataract extraction, a toxocara scar and tractional retinal detachment was discovered. The patients are so full of hope and though most cases result in success, it can be difficult to accept the less than ideal outcome.

One story from KSF is that of a married couple in their early 50 s. Both patients had bilateral dense white cataracts and had not seen each other or their children for 10 years due to their pathology. They were operated upon on the same day and saw one another for the first time in our unit when the dressing was removed. It was a very emotional event for patients and staff alike.

### Delayed presentations

**Fig. 10:**
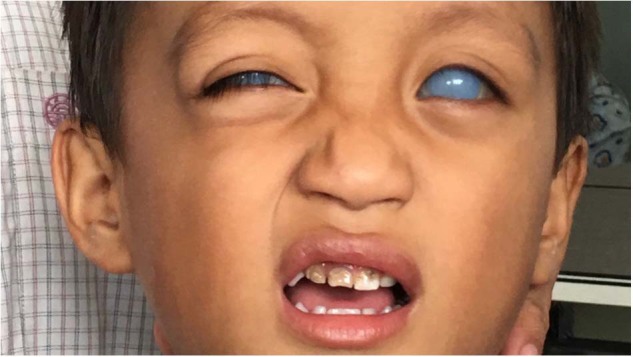
First presentations of congenital glaucoma.

As shown in Fig. 10, some patients present with ophthalmic problems such as congenital glaucoma and sky high pressures. In these cases it is often too late.

During this same clinic, a mother brought her newborn child to the KSF clinic. The mother told us that the baby had not opened her eyes since birth. On examination this was because the baby was bilaterally anophthalmic. This case was very difficult - having to explain to the mother that her newborn would never see. Sadly, due to cases of infanticide related to congenital defects, social services had to be contacted to protect the child from any harm. Delivering this sort of news to parents, via a translator and in a completely different cultural context, requires a lot of gentleness despite the buzz and extreme busyness of the clinic.

## Optometry

### Role of optometry

Optometry is a very valuable resource and the optometrists who have worked with KSF have performed a huge range of tasks from handing out donated glasses, performing refractions, reviewing patients in clinic, examining pre and post operative cases and delivering anaesthetic blocks.

The WHO estimates that 39% of global blindness is due to cataract and 18% is due to uncorrected refractive errors [5]. They have not commented upon presbyopia in this article but in Cambodia people simply cannot get reading glasses. In a country that does not have a social welfare system and to work means to feed one’s family, people are expected to work without being able to see. A seemingly simple treatable cause of low vision would not alter one’s life to such a degree in the UK.

A simple life changing task KSF carry out is the delivery of reading glasses to patients during village screening. This has a huge impact. Unfortunately, patients sometimes keep these glasses as a special item rather than something to use so KSF undertake patient education sessions on how to use them, the importance of their use and assurance that they can have more if needed.

Recently, Ace Vision (Boston, United States) helped run a fundraising event at the American Society of Cataract and Refractive Surgery (ASCRS) Annual Meeting where one company donated $50,000 USD for a 3D printer for glasses. This brings the cost of glasses to $1 USD each. This low price allows KSF to do even more to help tackle visual disability.

### The future of optometry in Cambodia

Optometrists help support the present ways KSF significantly reduce avoidable blindness in Cambodia through refractions, aiding cataract surgery and shortly they will be involved in the management of diabetic retinopathy.

Cambodia at present does not have any optometry training. KSF plans to start an optometry school. Training cannot be the same as a 4 year optometry degree program in the UK because this would exclude those who cannot afford 4 years of their time when they may have dependents of their own.

**Fig. 11:**
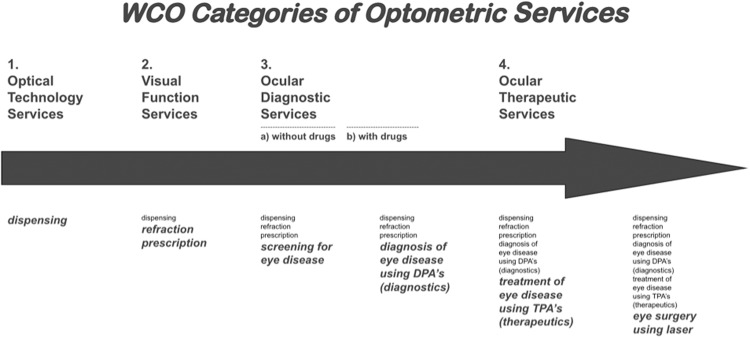
The different categories of optometric services.

**Fig. 12:**
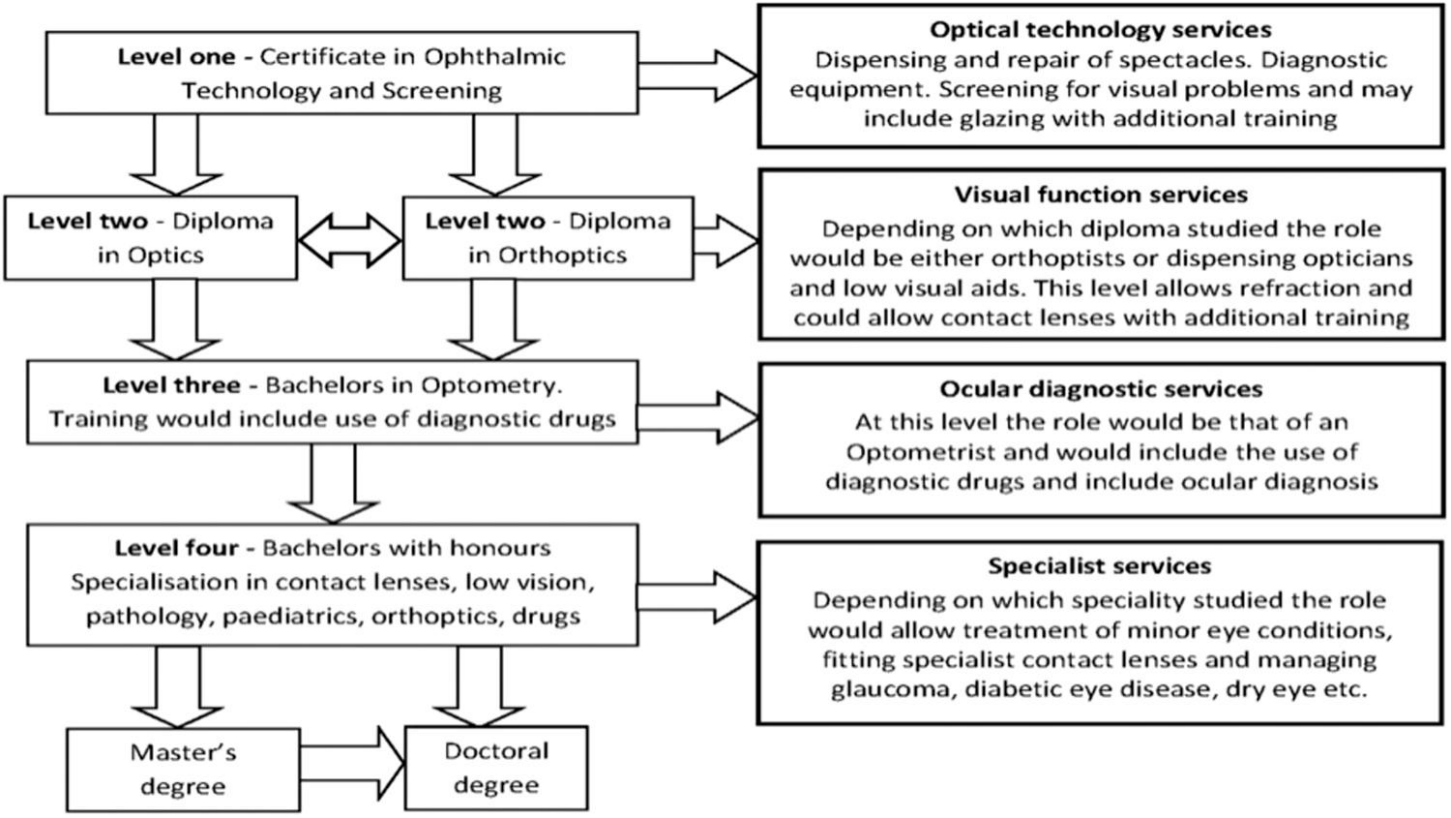
The potential optometry curriculum to be implemented by KSF in Cambodia.

Figure 11 shows the different categories of optometric services - glasses dispensing, visual function services, ocular diagnostic services and ocular therapeutic services. Working with Professor Shehzad Naroo from Aston University, KSF have mapped a potential optometry curriculum shown in Fig. 12. Each year would be a self contained level of achievement, allowing Cambodian people to train as optometrists with each year granting certification in a specific area of expertise. For example, at the end of year one, students would be basic refractionists. With each subsequent year completed, either as run-through or by leaving to work and then coming back, the course would progress to receiving a diploma in optics or orthoptics and then finally the equivalent of an optometry degree. This programme aims to be on par with an international optometry degree. We understand that a lot of optometrists may choose to leave the country once completing training but feel that they should be able to make those decisions regarding how best to progress themselves personally and professionally. We do expect that many trained with this programme will come back and work in Cambodia should they choose to leave for a period.

## Ophthalmology

### Role of ophthalmologists

KSF typically operate on 200 eyes a week during a mission. Junior ophthalmologists perform most of the clinical work; assessing patients preoperatively, consenting and listing for surgery and undertaking post-operative checks. They also perform anaesthetic blocks. At present cataract and pterygium surgery are the mainstays. KSF is recruiting for a full-time surgeon and thereafter will look to commencing glaucoma surgery and corneal graft surgery.

KSF only allows senior surgeons to operate due to the challenging nature of the cases. Furthermore, not all cases are suitable for phacoemulsification - some of the cataracts have splintered the stainless steel phaco tips. Different methods of surgery are employed such as small incision cataract surgery or intracapsular cataract surgery as and when required.

**Fig. 13:**
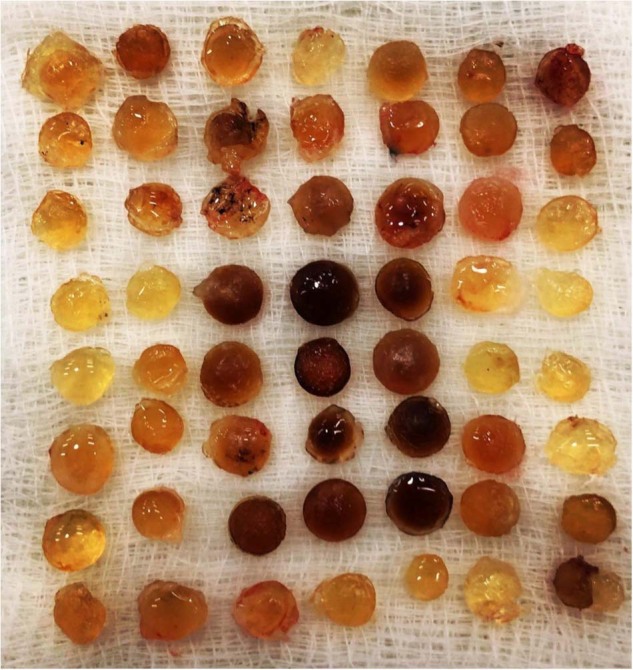
The output from one surgeon performing small incision cataract surgery in a single day.

Figure 13 shows the output from one surgeon performing small incision cataract surgery during one day. These cases are challenging and also high in volume - the highest number performed was 110 in one day from a single surgeon.

There are a also a high number of posterior polar cataracts. When operating, surgeons have to think on their feet - if something does not work one has to make safe, intelligent decisions. Most ophthalmologists are delighted with their time working with KSF; they have to think outside the box and undertake work that is not typical of their day-to-day hospital activities. So much so that it is not thought of as ‘work’ even though the numbers of patients being operated on is far in excess of what they are used to at home.

### Surgery in community versus based in one place

One of the decisions KSF made early on was where to base the clinic. The choice was between working as a mobile unit setting up in villages or as a static operating site. KSF decided that it would be best to have a static place in the capital, with good equipment and reliable resources.

### The future of Ophthalmology in Cambodia

Cambodia only trains 5 residents a year. Local politics prevent a reasonable number being trained and in previous years the official allocated places for residents was zero. Nepotism is also prevalent. To circumvent this, KSF have begun an ophthalmic residency program for Cambodian doctors which has been approved by the government. KSF does not just want to provide a cataract surgery service, it wants to help create a sustainable future for generations to come.

## Next steps

I have helped develop “GERSO” Global Education and Research Society of Ophthalmology. This is an online didactic training program. Part of this, which will be launched soon, is a section aimed at training ophthalmologists for the International Council of Ophthalmology curriculum which is primarily aimed at developing countries.

As with any charity, fundraising is always a problem. KSF works on donations and has set up charity fundraisers such as ‘Dining in the Dark’, an event held at the ASCRS Annual Meeting during which attendees dine blindfolded. However, fundraising remains a significant concern.

One of the ways that KSF raises money is to perform paid research. KSF runs trials on devices and IOLs and any funds raised pay for other patients to have free surgery. This sometimes means some patients are getting the next generation trifocal IOL lenses for free.

KSF plans to continue with our charity missions alongside continuing with all our plans above.

KSF takes over your life and though you may feel physically tired afterwards, you feel emotionally refreshed because you are doing something so meaningful. The gratitude received from the patients inspires you to do even more. KSF has been a lifeline for many patients and their families who rely on them to eat. In Cambodia, KSF does not intend to achieve perfect vision for patients - it is more about getting enough vision so patients can live independent lives.

There are many hurdles that KSF has overcome, and we are always amazed by the willingness of friends and colleagues to extend help and support – a testament to the undeniable importance of KSF’s work.
